# Software for selecting the most informative sets of genomic loci for multi-target microbial typing

**DOI:** 10.1186/1471-2105-14-148

**Published:** 2013-05-01

**Authors:** Matthew VN O’Sullivan, Vitali Sintchenko, Gwendolyn L Gilbert

**Affiliations:** 1 Centre for Infectious Diseases and Microbiology and Sydney Institute for Emerging Infections and Biosecurity, University of Sydney, Westmead Hospital, Hawkesbury Road, Westmead, NSW 2145, Australia

**Keywords:** Comparative genomics, Multilocus sequence typing, MVLA, Binary typing, Software, Microbial typing, MRSA, *Cryptococcus*, *Staphylococcus aureus*, *Streptococcus pneumoniae*

## Abstract

**Background:**

High-throughput sequencing can identify numerous potential genomic targets for microbial strain typing, but identification of the most informative combinations requires the use of computational screening tools. This paper describes novel software – Automated Selection of Typing Target Subsets (AuSeTTS) - that allows intelligent selection of optimal targets for pathogen strain typing. The objective of this software is to maximise both discriminatory power, using Simpson’s index of diversity (*D*), and concordance with existing typing methods, using the adjusted Wallace coefficient (*AW*). The program interrogates molecular typing results for panels of isolates, based on large target sets, and iteratively examines each target, one-by-one, to determine the most informative subset.

**Results:**

AuSeTTS was evaluated using three target sets: 51 binary targets (13 toxin genes, 16 phage-related loci and 22 SCC*mec* elements), used for multilocus typing of 153 methicillin-resistant *Staphylococcus aureus* (MRSA) isolates; 17 MLVA loci in 502 *Streptococcus pneumoniae* isolates from the MLVA database (http://www.mlva.eu) and 12 MLST loci for 98 *Cryptococcus* spp. isolates*.*

The maximum *D* for MRSA, 0.984, was achieved with a subset of 20 targets and a *D* value of 0.954 with 7 targets. Twelve targets predicted MLST with a maximum *AW* of 0.9994. All 17 *S. pneumoniae* MLVA targets were required to achieve maximum *D* of 0.997, but 4 targets reached *D* of 0.990. Twelve targets predicted pneumococcal serotype with a maximum *AW* of 0.899 and 9 predicted MLST with maximum *AW* of 0.963. Eight of the 12 MLST loci were sufficient to achieve the maximum *D* of 0.963 for *Cryptococcus* spp.

**Conclusions:**

Computerised analysis with AuSeTTS allows rapid selection of the most discriminatory targets for incorporation into typing schemes. Output of the program is presented in both tabular and graphical formats and the software is available for free download from http://www.cidmpublichealth.org/pages/ausetts.html.

## Background

Microbial strain typing schemes, with variable discriminatory powers, are increasingly applied to study long-term evolution, detect emergence of new or hyper virulent clones, identify outbreaks and track transmission chains. New high-throughput DNA sequencing methods identify hitherto unrecognised variation in the genomes of even closely related isolates, which is a valuable source of targets for use in new microbial typing schemes. These genotyping systems can be tailored to have discriminatory power appropriate for the purpose [1] but systematic assessment of the characteristics of potential targets is required to ensure the quality and reliability of the resulting typing scheme.

Existing typing systems involve interrogation of several genetic loci to determine sequence variation (e.g. multi-locus sequence typing, MLST), length polymorphisms (e.g. multi-locus variable number of tandem repeats analysis, MLVA) or the presence or absence of genetic targets (i.e. binary typing). Next generation sequencing technologies have yielded vast amounts of sequencing information for a wide variety of organisms, and bench top sequencers permit real-time sub typing of bacteria by sequencing small batches of bacteria in a matter of hours [2]. This has prompted some to advocate whole genome sequencing as a routine typing method [3], but limitations of data analysis and assigning cut-offs for relatedness mean that whole genome data is more commonly used to identify loci that may be useful to design informative typing systems [4]. A critical step in deciding which loci to incorporate into such typing systems is to estimate the discriminatory power and concordance with other typing systems that would be achieved with different combinations of loci.

The essential characteristics of a microbial typing system include appropriate discriminatory power for the research question being studied, consistency with both clinical epidemiology and established typing methods, stability, reproducibility, type ability, ease of use and interpretation, high throughput and low cost [5].

Discriminatory power is most frequently assessed using Simpson’s index of diversity (*D*), which gives the probability that isolates randomly selected from a population would differ using the typing method.

A number of indices can likewise be used to measure concordance between typing systems or between a typing system and epidemiologic classifications. The Wallace coefficient (*W*) estimates the probability that two isolates assigned the same type by the method under evaluation (M_1_) belong to the same type using the comparator method (M_2_). *W* is a directional measure; that is the results for the concordance of M_1_ with M_2_ are different from those of the concordance of M_2_ with M_1_.

When choosing targets identified by comparative genomics for incorporation into a new typing system, a good starting point is to select those that in combination give the most favourable results for these measures of discriminatory power and/or concordance using an existing collection of typed isolates. However, examination of every possible combination of candidate targets, individually, is often computationally expensive. For example, comparison of all possible subsets of 100 potential targets available for use in a typing system, to determine the most informative subset, would require 10^30^ calculations, which is beyond the capacity of standard computers. Therefore, alternative approaches are required. Software has been developed to interrogate informative single nucleotide polymorphisms (SNPs) in sequence based data (Minimum SNPs) but it is not designed to handle other forms of typing data [6,7]. Furthermore, while it can be used to identify SNPs, which are most predictive of a user-nominated sequence type, it does not consider overall measures of concordance between typing systems. We report here a new computational approach selecting the most informative sets of genomic loci for multi-target microbial typing and discuss its application to different typing methods for pathogenic bacteria and fungi.

## Implementation

In constructing an approach for interrogating combinations of targets, which are either binary and/or multistate (where a target can assume any of >2 possible values), we developed a heuristic based on the stepwise accumulation of informative targets. Here ‘informative’ means the combination of targets producing either the greatest discriminatory power or the greatest concordance with existing typing methods (as selected by the user). This heuristic assumes that the most informative combination of *n* + 1 targets includes the most informative combination of *n* targets as a subset. While this assumption may not always hold true, it vastly reduces the number of combinations that need to be examined to determine the maximally informative subset of targets and it can be confirmed *post-hoc* for a given dataset.

AuSeTTS (Automated Selection of Typing Target Subsets) is a software program designed to analyse a large array of typing data for a panel of isolates and determine the optimal combination of typing targets to maximise discriminatory power and/or concordance measures for a specified subset size. The analysis can be performed with (heuristic search) or without (exhaustive search) the heuristic described above. The software was written in Microsoft Visual Basic for Excel (2010); it is available for free download from http://www.cidmpublichealth.org/pages/ausetts.html and also accompanies this paper (Additional file 1).

The input data consist of a table of typing results with the targets in columns and the isolates in rows. Each cell represents the result for a given target in a given isolate and is expressed as character-based data (for example 0 or 1 for binary data, allele numbers for MLST or numbers of repeats for MLVA data). One or more columns can be specified as the comparator typing method for calculating measures of concordance and typing results can be represented in the dataset multiple times by providing numbers of isolates for each row in a specified column. Non-informative targets (i.e. which have the same result for every isolate or are completely concordant with a second target) are automatically removed from the set before analysis.

**Figure 1:**
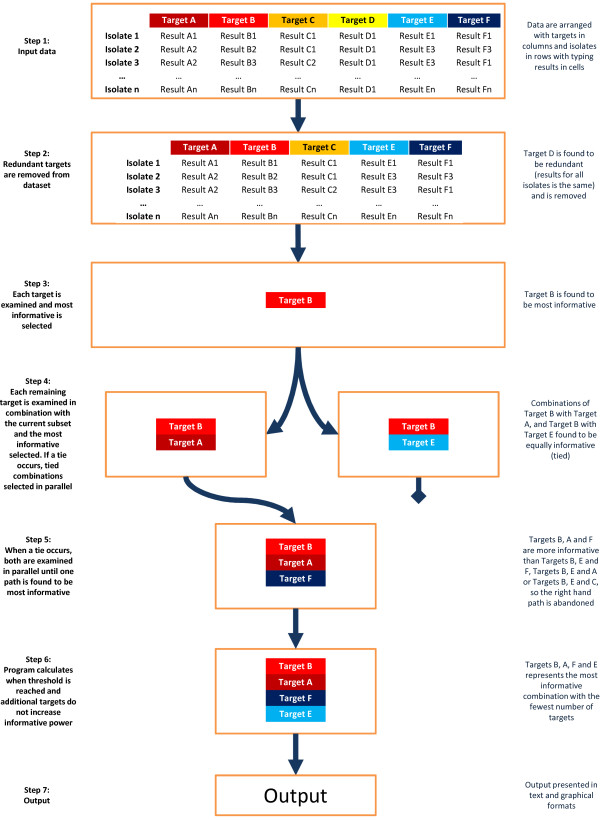
Schematic overview of iterative assessment of typing targets conducted by AuSeTTS (heuristic search).

**Figure 2:**
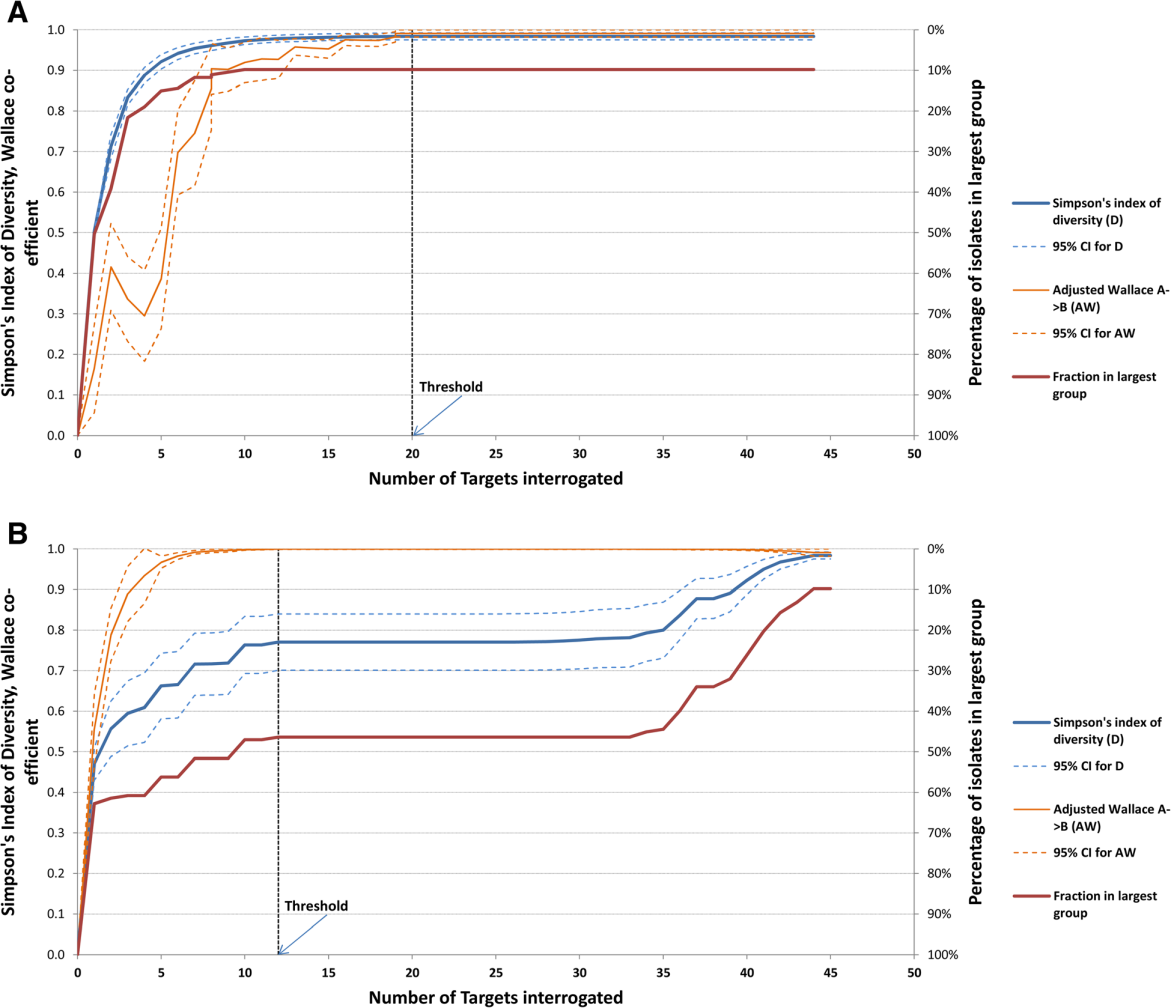
**AuSeTTS graphical output.** (**A**). Relationship between the number of target loci and the discriminatory power of molecular subtyping. Results for analysis of MRSA binary typing data. The maximum Simpson’s Index of Diversity was achieved with a combination of 20 targets. (**B**). MRSA binary typing data analysis to maximise the Wallace coefficient. Maximum concordance of binary type to predict MLST was achieved with 12 binary targets, with an *AW* value of 0.994.

Using the heuristic search, the software initially ranks each target by their individual discriminatory power or concordance. It then examines all other targets in combination with the most informative target(s) to identify the most informative combinations of two targets. Further targets are then added iteratively until the whole dataset has been examined. When a ‘tie’ between combinations is encountered each of the tied combinations continue to be considered, with additional targets being added until the ties are broken. Once the ties are broken, the less informative combination(s) are abandoned. A ‘threshold’ is ultimately determined: the number of targets, beyond which adding more targets does not further increase discriminatory power or concordance. Figure 1 presents a schematic overview of the program. The output is a list of targets for each subset size that maximise discriminatory power or concordance, with the results of these measures and 95% confidence intervals. The information is also presented graphically (Figure 2).

Using an exhaustive search, the user specifies the number of targets to be included (the subset size). The software then examines every possible combination of targets producing a subset of this size and calculates the discriminatory power (and, if specified, the concordance measures). The combinations with the highest achievable discriminatory power are returned, along with 95% confidence intervals. The exhaustive search gives a definitive result that is not dependent on the heuristic. It may not be feasible to examine very large datasets with an exhaustive search: on testing, examining a subset of 5 binary targets from a dataset of 20 targets for 100 isolates (15,504 possible combinations) took 20 seconds, while doubling the number of targets to 10 from the same dataset increases the number of combinations to be examined by more than 10-fold which led to a corresponding increase in the computing time. Thus the problem using the exhaustive search becomes NP-complete for very large datasets, and the heuristic approach becomes necessary.

### Formulas

The formula used for calculating *D* was as follows:

$$D=1-\frac{1}{N\left(N-1\right)}\sum_{j=1}^{S}n_{j}\left(n_{j}-1\right)$$

Where *N* is the number of isolates in the sample population, *S* is the number of distinct types identified in the population and *n*_*j*_ is the number of isolates of the type *j*[8]. The following formulas have been developed for calculating confidence intervals for *D*[9,10]:

$$\begin{array}{l} \sigma^{2}=\frac{4}{N}\left[\sum {\left(\frac{n_{j}}{N}\right)}^{3}-{\left({\sum \left(\frac{n_{j}}{N}\right)}^{2}\right)}^{2}\right]\\ \;\mathrm{CI}=\left\lfloor D-2\sqrt{\sigma^{2}},D+2\sqrt{\sigma^{2}}\right\rfloor \end{array}$$

Where σ^2^ is the variance and *CI* is the approximate 95% confidence interval. This formula used for variance is a large sample approximation; a non-approximated formula for variance has also been described [10].

To calculate *W*, the typing results for both methods for each isolate in the data set must be examined against those for every other isolate in the data set to see if they match or are discordant. The formula used for *W* is given by [11]:

$$W_{\left(M_{1},M_{2}\right)}=\frac{\alpha }{\alpha +b}$$

Where *a* is the number of instances where two isolates of the same type by method *M*_*1*_ are of the same type by method *M*_*2*_, while *b* is the number of instances where two isolates of the same type by method *M*_*1*_ are of a different type by method *M*_*2*_. The Adjusted Wallace coefficient (*AW*) incorporates an adjustment to account for concordance that may occur by chance alone. The formula for *AW* is given by [12]:

$${\mathrm{AW}}_{\left(M_{1},M_{2}\right)}=\frac{W_{\left(M_{1},M_{2}\right)}+D_{\left(M_{2}\right)}-1}{D_{\left(M_{2}\right)}}$$

Where *D*_*(M2)*_ is the Simpson’s index of diversity of the dataset using typing method *M*_*2*_. In addition, the Rand (*R*), adjusted Rand (*AR*) and the approximate 95% confidence intervals of *AW* are also calculated [12,13]. The analytical confidence interval calculations for *W* may not be valid for *W* values of <0.5. An alternative method for calculation of confidence intervals for these measures of congruence is to use Jackknife resampling [14], for which an online tool is available [15].

Confidence intervals are provided for the purposes of comparison of results with other typing methods. However, in the algorithm, only the point estimates of *D*, *AW*, or *AR,* without confidence intervals, were used to determine the most informative values of each combination of targets. This approach reduces the complexity of the heuristic and, hence, the computation time required but the results relate only to the input dataset. The optimal combination of targets may therefore be different for larger sample sizes or samples from different populations of the same microbial species.

## Results and discussion

### Validation

To examine the robustness of the assumption that targets may be added in a stepwise fashion while maximising the parameter of interest (heuristic search), random datasets were generated and tested using both search types. These random datasets were defined by varying a) the number of targets, b) the number of different states each target could assume, c) the number of strain types and d) the number of isolates distributed (unevenly) amongst the strain types.

For each dataset, a heuristic search was used to calculate the threshold subset size. The heuristic search result for a subset of one target less than the threshold was compared with an exhaustive search result specifying the same sized subset. If the resulting maximum parameter value, using the exhaustive search was the same as that of the heuristic search, the heuristic was considered to be valid. If the maximal parameter value achieved by the heuristic search was less than that using the exhaustive search, the heuristic was considered not to have held. 25600 randomly generated datasets were examined for each of the 5 parameters of interest. The heuristic was valid in 79.4% (95% confidence interval 79-80), 98.2% (98-99), 83.4% (0.83-0.84), 92.9% (92-93) and 93.6% (93-94) of random datasets for *D, AW*_(A>B)_, *AW*_(B>A)_, *R* and *AR*, respectively.

Factors associated with failure of the heuristic to identify the combination of targets that maximised *D* included: a value of *D* between 0.90 and 0.96, and a larger number of targets analysed. It performed best when the maximum *D* of the whole dataset was 1 (87.8% 95% CI 87-89). The number of strain types, the number of isolates in the dataset and the number of states each target could assume did not influence the likelihood of the heuristic being valid.

The heuristic performed well for all four concordance measures. Factors associated with a lower likelihood of the heuristic being valid for concordance measures included an increasing number of targets in the dataset, *D* value of the dataset between 0.9 and 0.96, examination of a subset of close to half of the total number of targets and, for *AW*_(A>B)_, a maximum *AW* value between 0.1-0.35.

Full details of the validation are available in the supplementary material (Additional file 2).

### Application

The software was used to analyse different forms of microbial typing data generated by well-validated methods, specifically, binary typing data for *Staphylococcus aureus*[16-18], MLVA for *Streptococcus pneumoniae*[19] and MLST for *Cryptococcus* spp. [20,21]*.*

### Selection of targets for *Staphylococcus aureus* strain typing

Typing results for 51 binary targets in 153 methicillin-resistant *S. aureus* (MRSA) isolates (42 well characterised reference isolates and 111 clinical isolates from our institution) were available from previous experiments in our laboratory [16-18]. The targets comprised: 13 toxin genes [17], 16 phage-derived open reading frames [18] and 22 SCC*mec* elements [16] which had been interrogated using multiplex-PCR reverse line blot assays [22,23].

The maximum *D* value of binary typing with all 51 targets for this collection of MRSA isolates was 0.984 (95% confidence interval 0.975-0.992). AuSeTTS heuristic search showed that this could be achieved with a subset of 20 binary targets, while a subset of just 7 targets achieved a *D* value of 0.954 (0.941-0.967) (Figure 2A). When used to predict MLST (which had been determined by either the conventional [24] or SNP-based [25] methods for all 153 isolates), a maximum Adjusted Wallace coefficient of concordance (*AW*) of 0.9994 (0.999-1.000) was achieved with 12 targets (Figure 2B). One binary type consisted of two isolates with different MLST (which were single-locus variants). Isolates within each of the remaining binary types all belonged to one MLST type.

This data was used to develop a novel 19-target binary typing system for MRSA [26].

### Selection of targets for *Streptococcus pneumoniae* strain typing

Results of MLVA typing, using 17 loci, for 1449 *Streptococcus pneumoniae* isolates (representing 906 possible MLVA types) were available from the MLVA online database (http://www.mlva.eu) [19] for analysis by AuSeTTS. A maximum *D* of 0.997 (0.997-0.998) was achieved with all 17 loci but only 4 targets were required to achieve a *D* value of 0.990 (0.988-0.991), which divided the isolates into 438 MLVA types.

A subset of the isolates for which MLVA results were available also had been serotyped (537 isolates representing 43 serotypes and 398 MLVA types), and these we used to determine the combination of MLVA loci which could best predict the serotype. A maximum *AW* of 0.899 (0.857-0.942) for serotype was achieved using 12 of the MLVA loci. This particular combination of 12 targets divided the dataset into 370 MLVA types, 352 of which contained only one serotype, while 15 contained two, two contained one and one MLVA type represented by 6 isolates harboured 5 different serotypes.

A similar analysis was performed with MLST data which were available for 96 of the isolates consisting of 27 sequence types (ST) and 77 possible MLVA types. A maximum *AW* of 0.963 (0.943-0.983) for MLVA to predict ST was achieved with 9 targets which divided the 96 isolates into 60 MLVA types. One MLVA type consisted of 3 isolates with 3 different MLST types. All other MLVA types consisted of isolates with matching MLST types.

### Selection of targets for *Cryptococcus* species strain typing

Twelve MLST loci for 98 *Cryptococcus* spp. isolates from a previously published study [21] were examined using AuSeTTS. Eight of the 12 MLST loci provided a maximum *D* of 0.963 (0.945-0.981) for *Cryptococcus* spp.in a heuristic search. The exhaustive search, specifying a subset size of seven loci, indicated the same maximal *D* value could be achieved with only seven loci; i.e. for this dataset, the heuristic was invalid but the most informative combination of targets could still be identified using an exhaustive search. This analysis was used, in part, to determine the recommended targets for an international consensus protocol for MLST typing of *Cryptococcus* spp. [27].

### Discussion

AuSeTTS has been successfully applied to develop typing schemes for MRSA [26] and *Cryptococcus* spp. [27] and would be useful to assess the discriminatory power of combinations of candidate targets for typing systems for other pathogens. It can be used for a wide range of data types, but for interrogation of informative SNPs, we recommend Minimum SNPs, which has been designed specifically for this purpose [6,7]. Minimum SNPs should be used to examine input data in the form of multiple sequence alignments. AuSeTTS can also be used to examine the level of concordance between results produced using subsets of candidate targets and those of existing phenotyping or genotyping methods or with epidemiologic classifications. Minimum SNPs does provide some functionality with regard to concordance measures (the “not-N” mode), but does not calculate the Wallace or Rand coefficients or confidence intervals for the adjusted Wallace coefficient.

While the algorithm used in the heuristic search may not always provide a definitive result for the minimum subset size required for the maximal *D* value, it will be correct in the majority of cases. For smaller datasets, an exhaustive search can easily be undertaken to confirm the validity of the heuristic. This is particularly recommended if the dataset has several features that were associated with a higher likelihood of the heuristic being invalid, such as low maximum *D* values, a threshold value close to 50% of the total number of targets, a number of states each target can assume of <8 and a large number of unique strain types. A worked example demonstrating the use of AuSeTTS (Additional file 3) using a sample dataset (Additional file 4) accompany this paper.

## Conclusions

Computerised analysis with AuSeTTS enables rapid, automated identification of the most informative targets for incorporation into novel molecular typing schemes for bacteria and fungi. Discriminatory power and concordance, while important, are only two of the many parameters that need to be considered when developing a new molecular typing technique. Reproducibility, stability, ease of use, ease of interpretation, throughput and cost are additional measures that require thorough assessment and comparison with existing methods during development and evaluation of novel typing techniques [5].

## Availability and requirements

**Project name:** AuSeTTS

**Project home page:**http://www.cidmpublichealth.org/pages/ausetts.html

**Operating system(s):** Microsoft Windows

**Programming language:** Visual Basic for Applications

**Other requirements:** Microsoft Excel for Windows

**License:** Unrestricted Freeware

## Abbreviations

AR: Adjusted Rand coefficient of concordance; AW: Adjusted Wallace coefficient of concordance; AW(A>B): Adjusted Wallace coefficient of concordance for target combinations to predict the reference partitions; AW(B>A): Adjusted Wallace coefficient of concordance for the reference partitions to predict target combinations; D: Simpson’s index of diversity; MLST: Multilocus sequence typing; MLVA: Multilocus variable number of tandem repeats analysis; PCR: Polymerase chain reaction; SNPs: Single nucleotide polymorphisms; W: Wallace coefficient of concordance.

## Competing interests

The authors declare that they have no competing interest.

## Authors’ contributions

MOS developed the software, performed molecular typing on *S. aureus* isolates, conducted the data analysis and prepared the manuscript. GLG and VS provided expert guidance and edited the manuscript. All authors read and approved the final manuscript.

## Authors’ information

MOS is a clinical microbiologist, infectious diseases physician and was recently awarded a PhD on the topic of applied molecular typing in hospital infection control. VS is a clinical microbiologist whose research interests include molecular epidemiology of pathogens with epidemic potential and infectious diseases informatics. GLG is a clinical microbiologist and professor of infectious diseases whose interests include public health microbiology and hospital infection control.

## Supplementary Material

Additional file 1 The AuSeTTS software file. Click here for file

Additional file 2 The full description of the heuristic search validation. Click here for file

Additional file 3 **A worked example using the dataset in Additional file **4**.** Click here for file

Additional file 4 Sample AuSeTTS dataset. Click here for file
