# Supplementary material for: Software for selecting the most informative sets of genomic loci for multi-target microbial typing
Source: BMC Bioinformatics. 2013 May 1;14:148. doi: 10.1186/1471-2105-14-148 (PMC3660239; doi:10.1186/1471-2105-14-148)

AuSeTTS validation details

The heuristic was validated by processing multiple random datasets for each of the parameters of interest. For each dataset, the heuristic mode was first used to establish the “threshold” number of targets which, in combination, maximised the parameter of interest. The maximum parameter value by the heuristic method using a combination size which was one less than the threshold was noted. Next, the exhaustive mode was utilised on the dataset, again, examining a combination size of one less than the “threshold”. If the exhaustive mode yielded a maximum parameter value which was the same as that achieved by the heuristic for that combination size, the heuristic was recorded as being valid for that dataset, otherwise it was said to be invalid.

For each of the validations of *D*, $AW_{(A\text{->}B)}$, $AW_{(B\text{->}A)}$, *R* and *AR*, a new set of 25,600 random datasets was generated.. Each of the four input variables for each dataset could assume one of four values (table 1) and 100 datasets for each unique combination of four values was generated ($100 \times 4^4 =$ 25,600 datasets in total for each parameter).

**Random dataset input variable**	**Possible values**			
Number of targets	4	5	6	7
Number of states each target could assume	2	3	4	5
Number of unique strain types*	10	12	14	16
Ratio of number of isolates to the number of unique strain types in each dataset*	1	2	3	4

Table 1. Parameters used to generate the random datasets. *These two parameters were multiplied to give the number of isolates within each random dataset. As a result the number of isolates could be one of 16 values ranging 10 through to 64. Datasets with 10-16 isolates therefore had one isolate per strain type.

The influence of various dataset parameters on the likelihood that the heuristic was valid was examined, and is outlined in the five figures below.

Figure 1. Validation of heuristic for Simpson's index of diversity. Error bars indicate 95% confidence intervals.

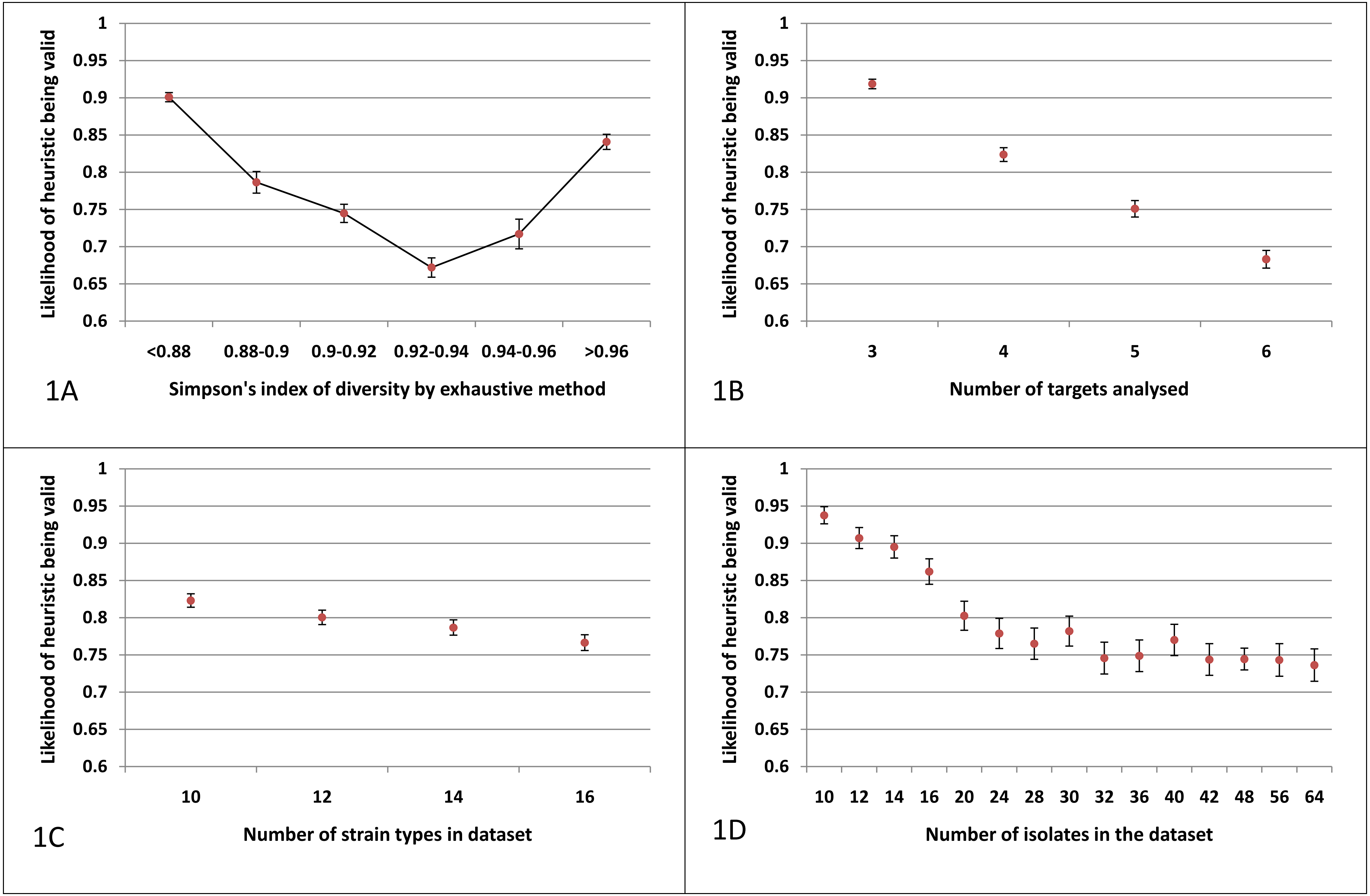

Figure 1 (continued). Validation of heuristic for Simpson's index of diversity.

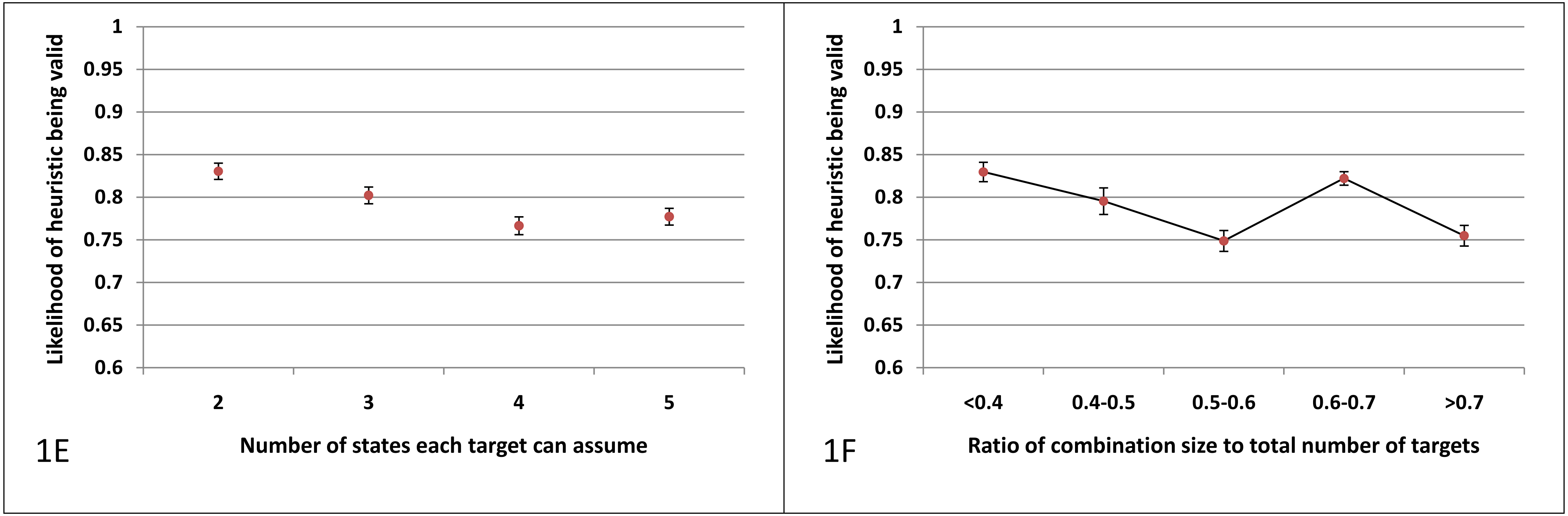

Figure 2. Validation of heuristic for the adjusted Wallace coefficient (A>B). Error bars indicate 95% confidence intervals.

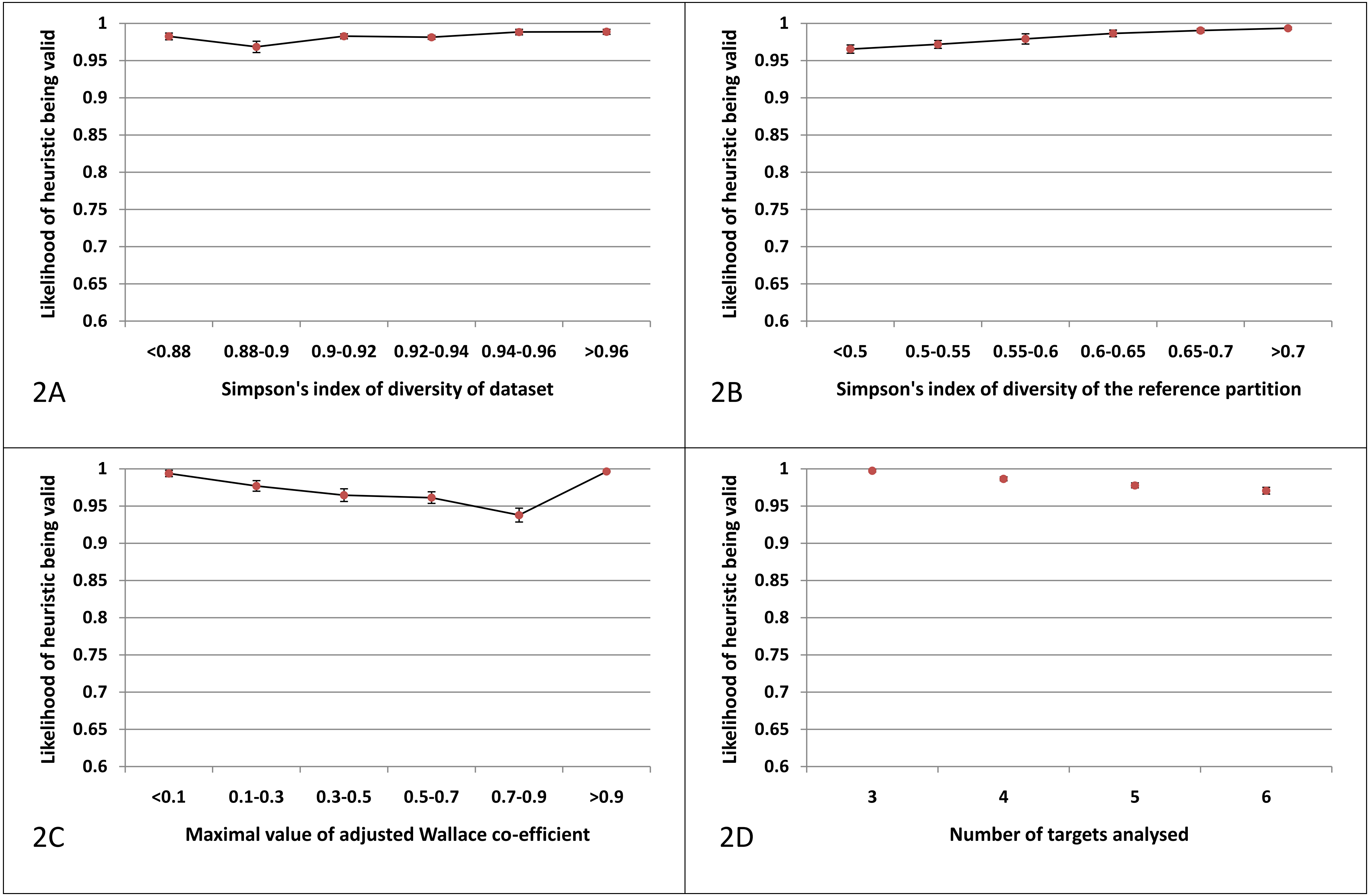

Figure 2 (continued). Validation of heuristic for the adjusted Wallace coefficient (A>B).

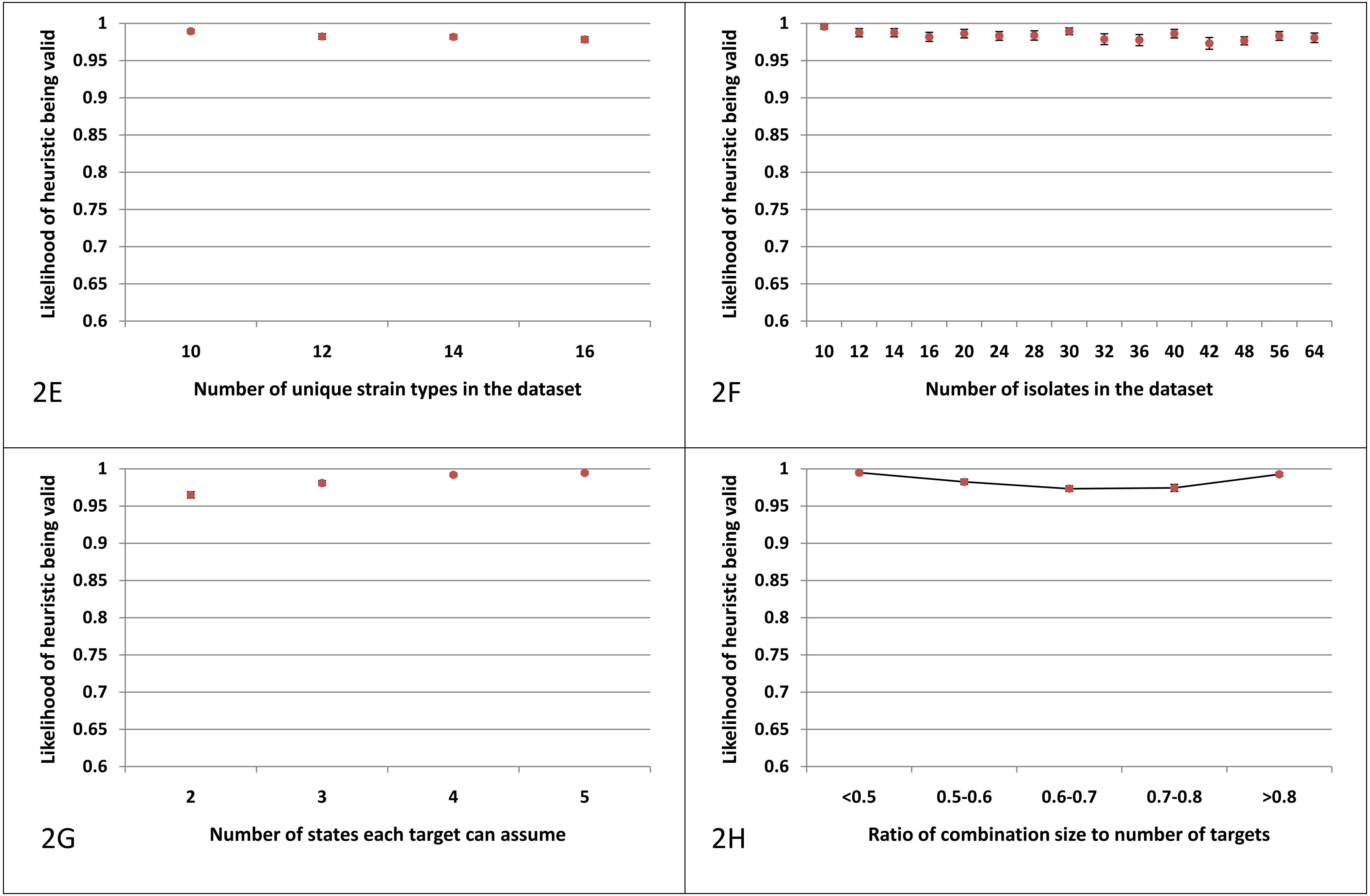

Figure 3. Validation of heuristic for the adjusted Wallace coefficient (B>A). Error bars indicate 95% confidence intervals.

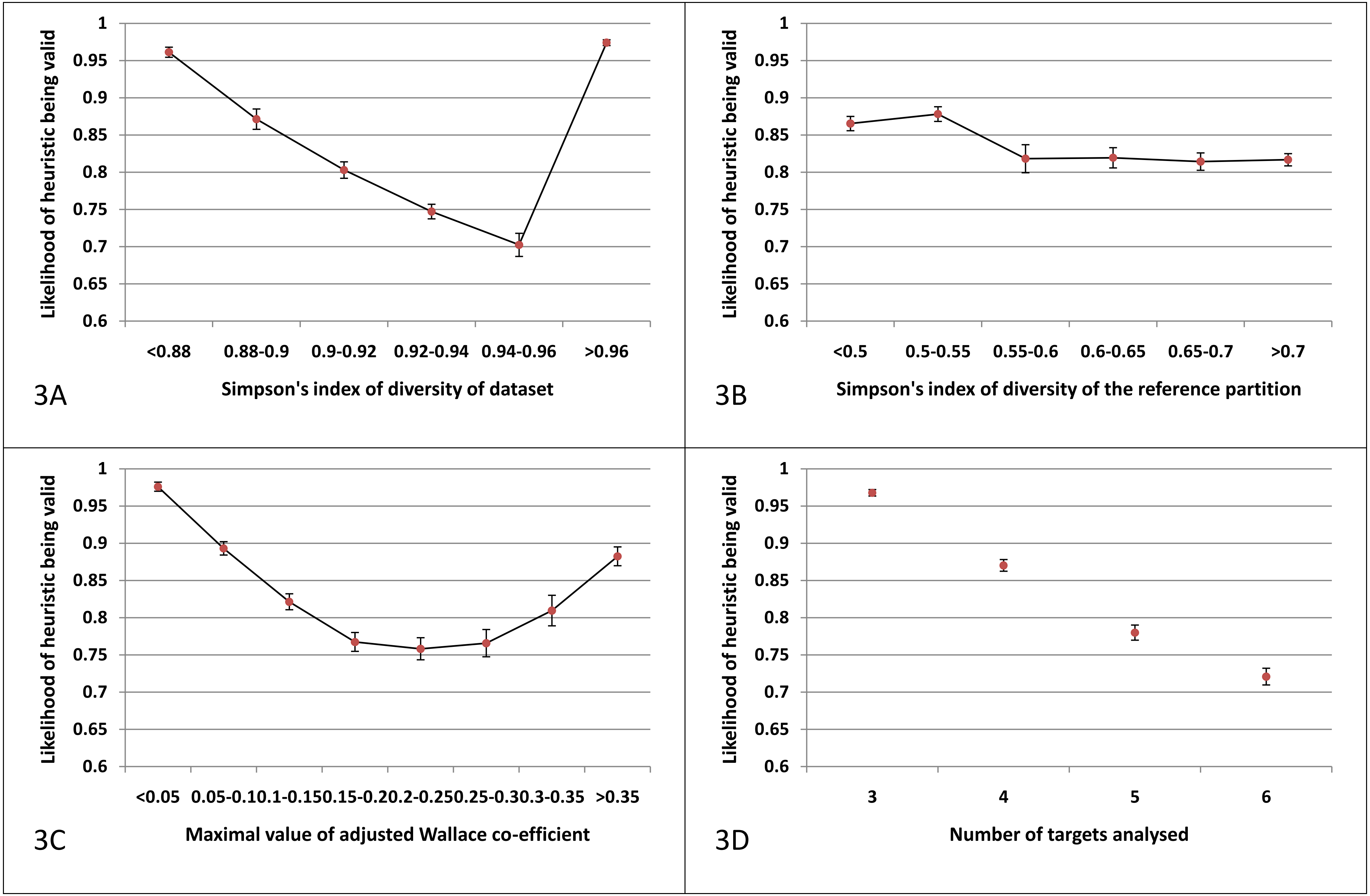

Figure 3 (continued). Validation of heuristic for the adjusted Wallace coefficient (B>A).

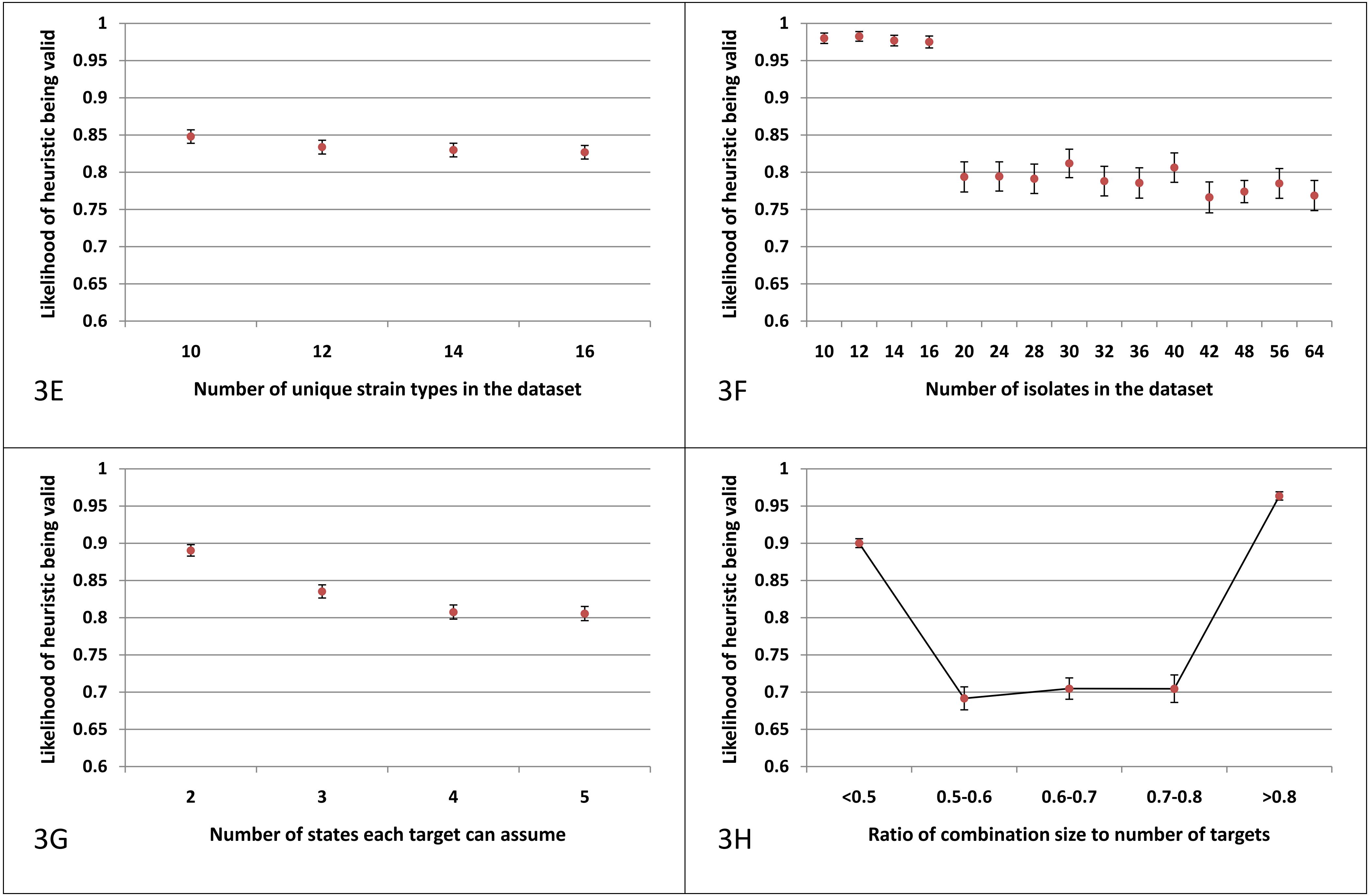

Figure 4. Validation of heuristic for the Rand coefficient. Error bars indicate 95% confidence intervals.

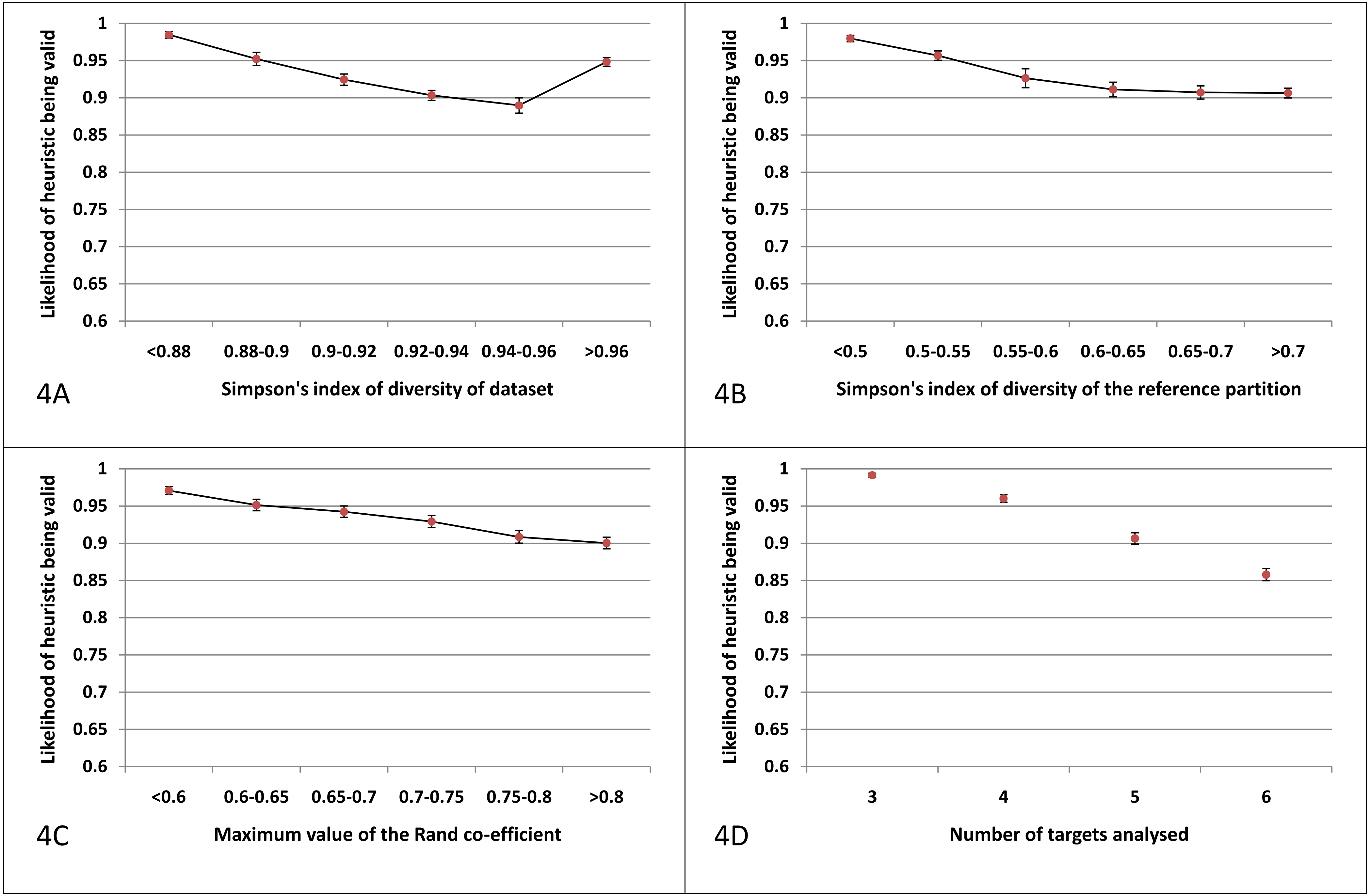

Figure 4 (continued). Validation of heuristic for the Rand coefficient.

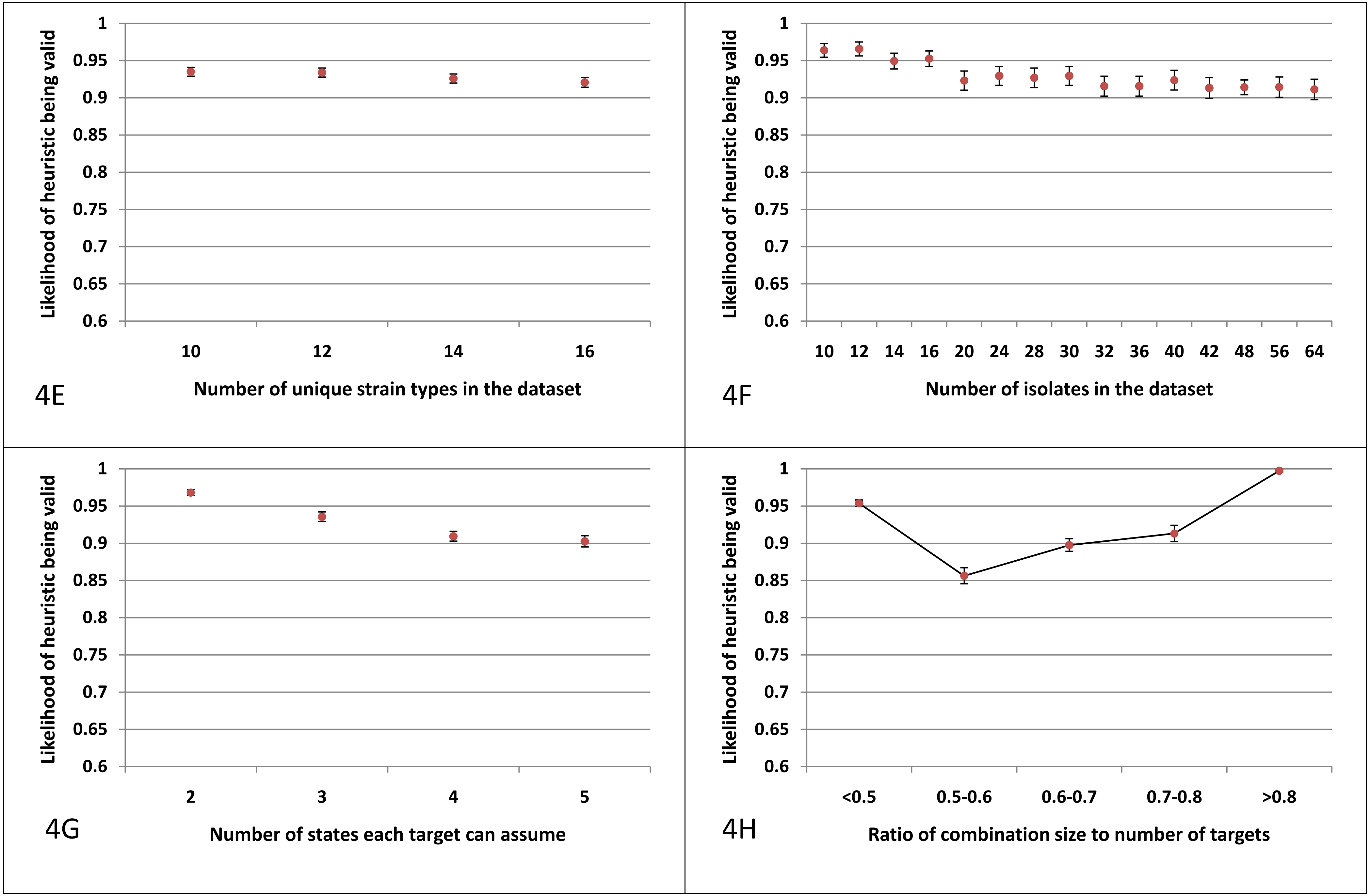

Figure 5. Validation of heuristic for the adjusted Rand coefficient. Error bars indicate 95% confidence intervals.

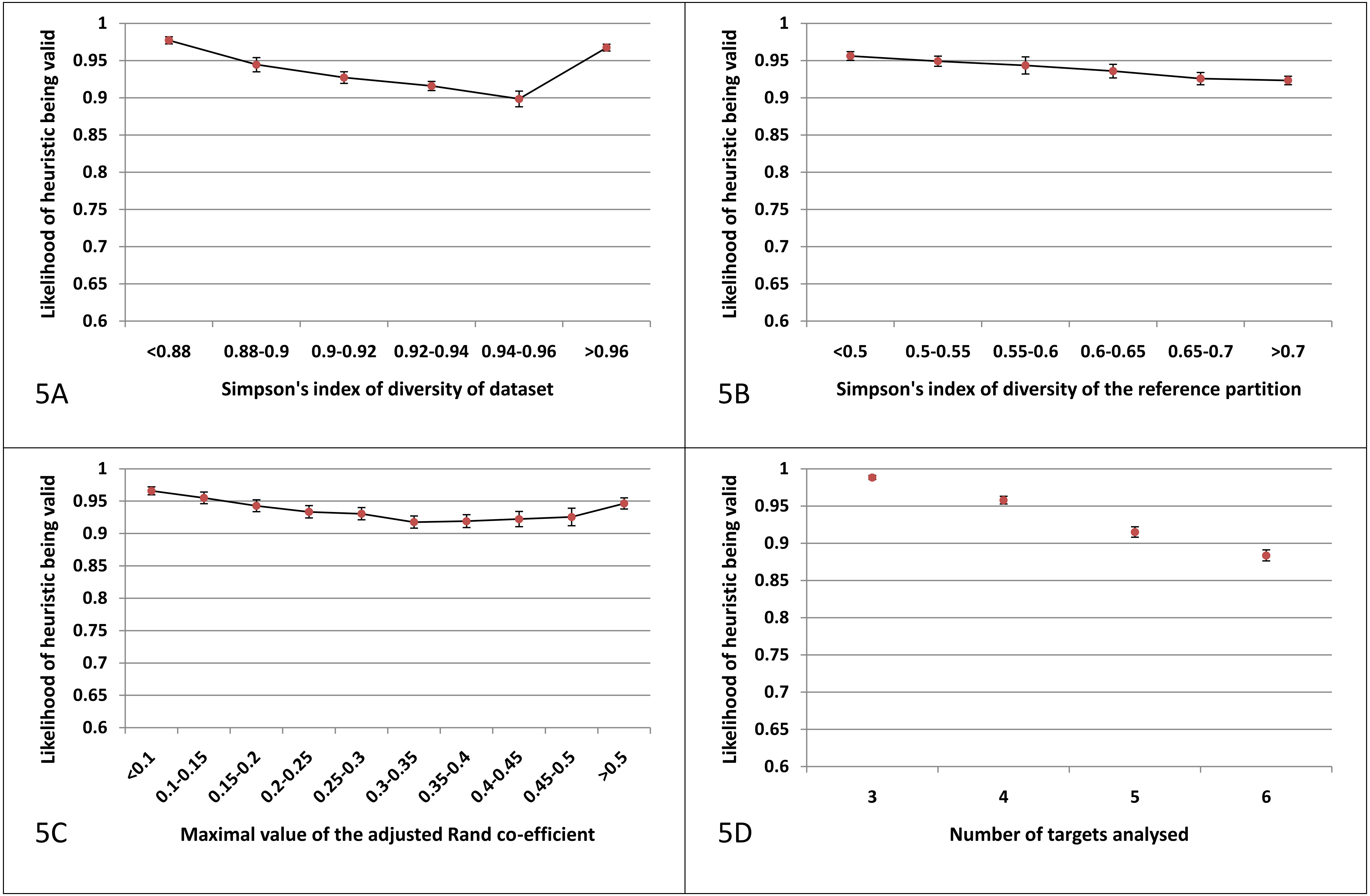

Figure 5 (continued). Validation of heuristic for the adjusted Rand coefficient.

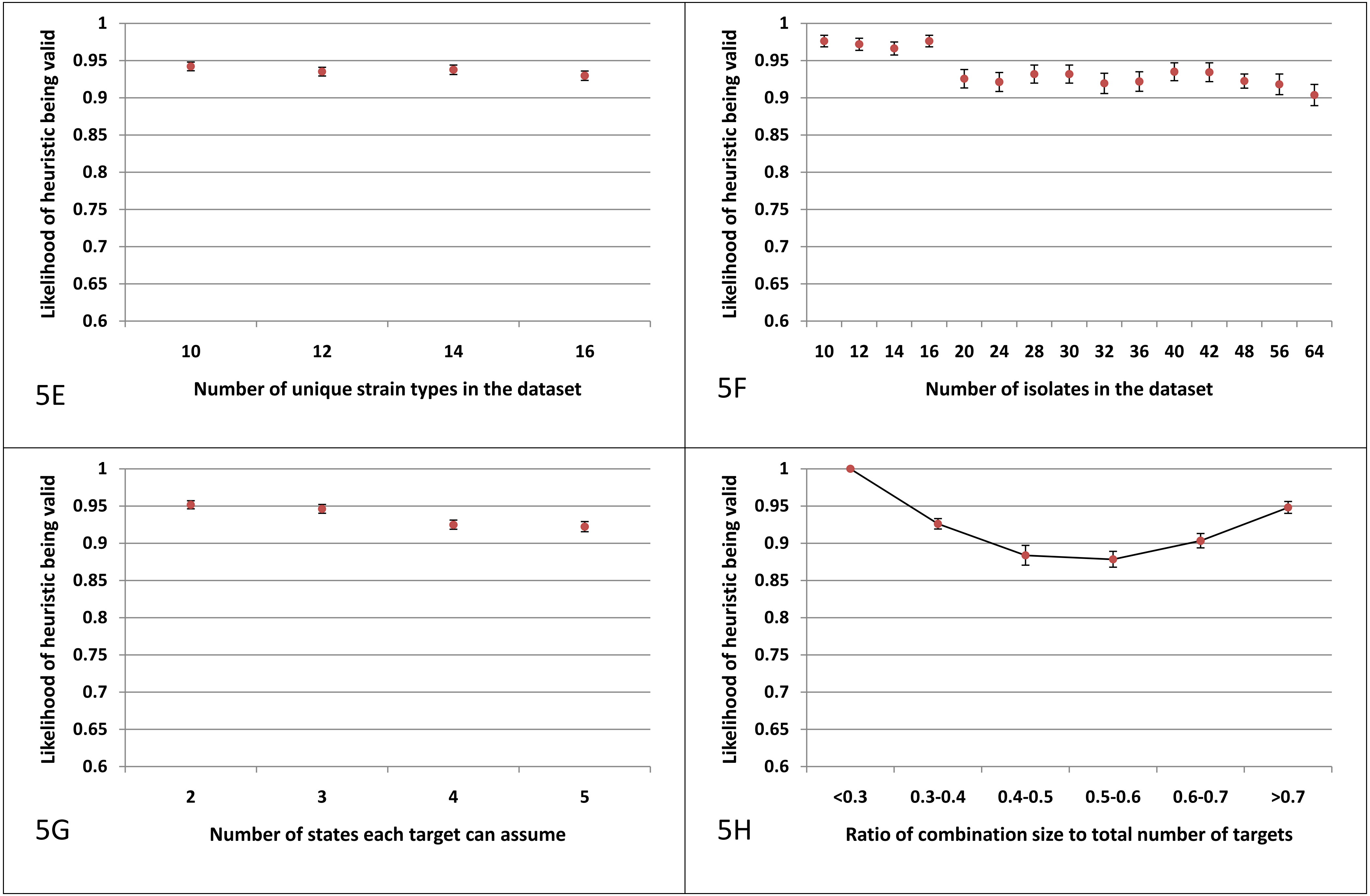

Supplement: Additional file 2 — The full description of the heuristic search validation. [file 1471-2105-14-148-S2.pdf]
