# Supplementary material for: Software for selecting the most informative sets of genomic loci for multi-target microbial typing
Source: BMC Bioinformatics. 2013 May 1;14:148. doi: 10.1186/1471-2105-14-148 (PMC3660239; doi:10.1186/1471-2105-14-148)
Supplement: Additional file 3 — A worked example using the dataset in Additional file 4. [file 1471-2105-14-148-S3.pdf]

AuSeTTS – Automated Selection of Typing Target Subsets

Worked Example

Step 1: Download Files

Download the files “AuSeTTSv6.xls” and “Sample AuSeTTS dataset.xls” to your computer

Step 2: Upload data

Open the file “AuSeTTSv6.xls”.

You may get a prompt to enable macros. Macros must be enabled for AuSeTTS to run:

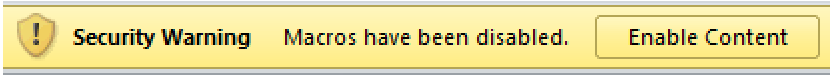

You will be prompted to load typing data:

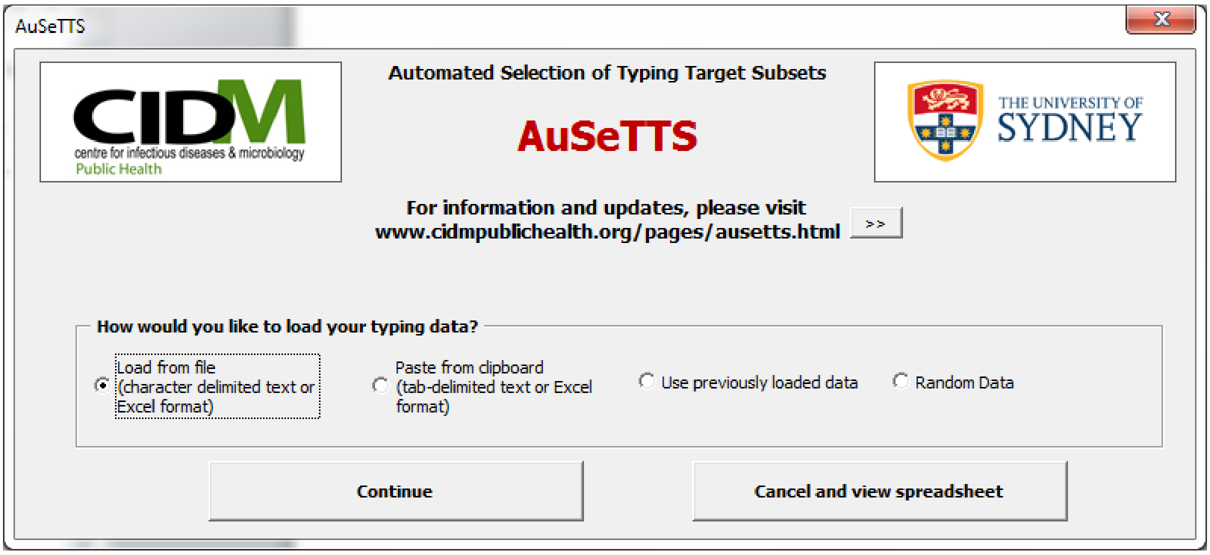

Choose “Load from file” and click continue.

> *Using the other options, you can also paste data from the clipboard, use the data already saved in the AuSeTTS file or create a random dataset. If you would like to view the results of an previously performed analysis that has been saved in the AuSeTTS file, click “Cancel and view spreadsheet”.*

Browse to the file “Sample AuSeTTS dataset.xls” on your computer and click open. You are prompted to choose the worksheet in the file that contains the typing data. Choose “Sheet 1” and click OK. The data is then loaded:

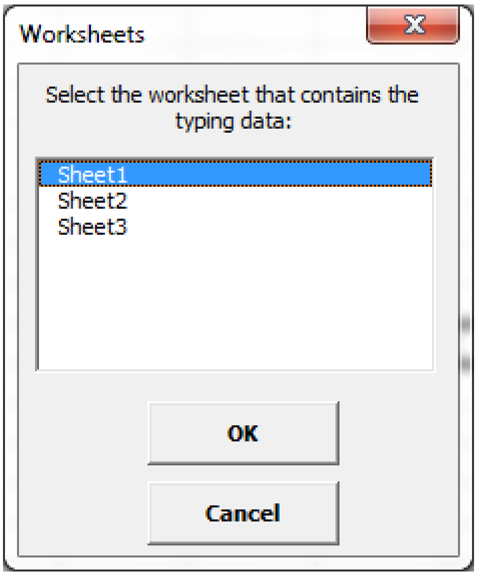

Step 3: Define fields

The targets in the dataset are listed in 4 windows. Highlight “MLST” in the list of “Target(s) defining partitions”. Concordance between different combinations of the binary targets and MLST will be calculated during the analysis.

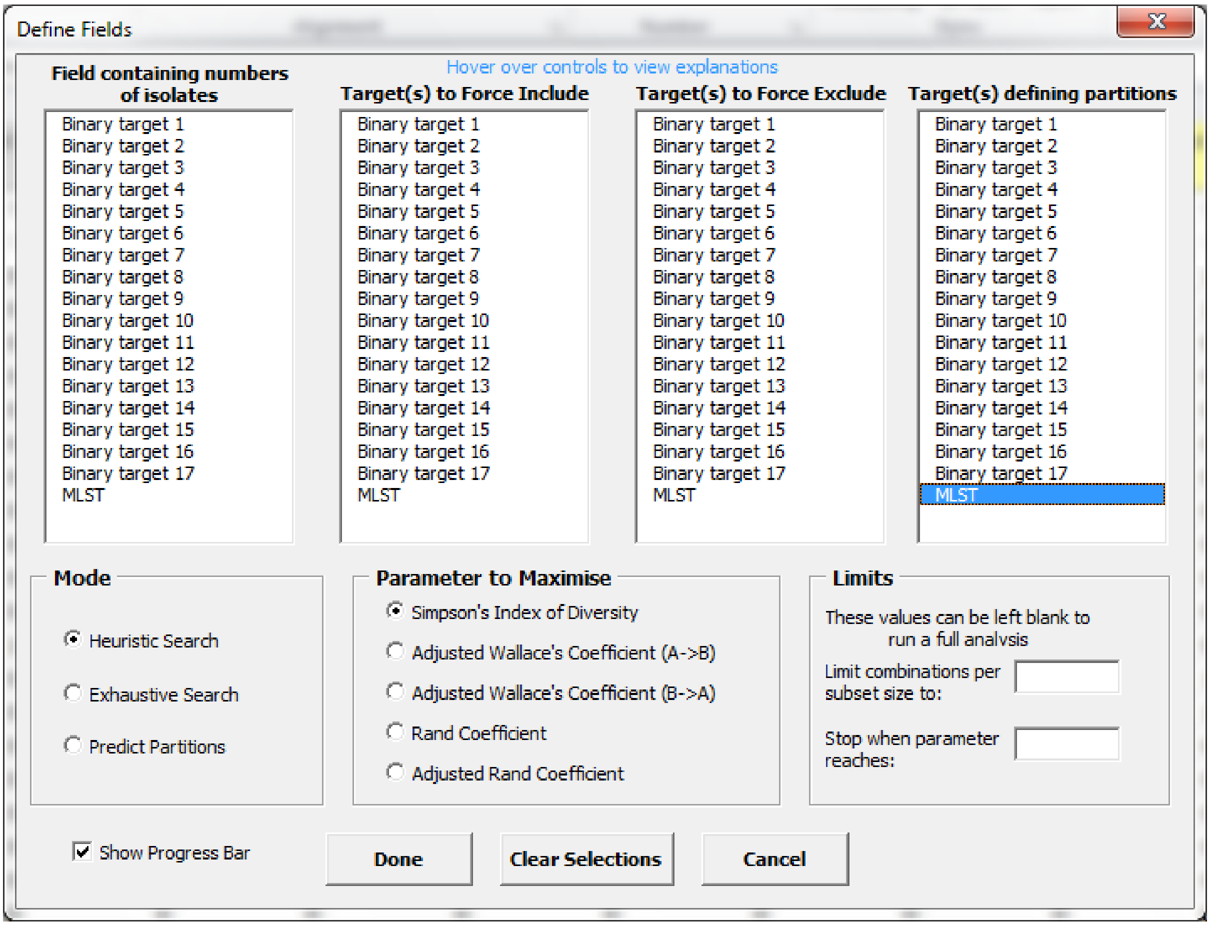

The window "Field containing number of isolates" is used where there is a single row in the dataset for each strain type, but you would each strain type to represent multiple isolates. In this case the data set is formatted as one row per isolate, so no field containing the number of isolates needs to be specified.

If there are particular target(s) of interest, these can be force included in the dataset using the second window. Force included targets will be automatically included in any combinations that are analysed.

Targets can be excluded from the analysis by selecting them in the third column. Targets selected in the first or last windows are automatically excluded.

Under "Mode" choose "Heuristic search". This will be a quick search of the dataset to find the combination of targets which appears to be most informative.

Exhaustive search is used to examine every possible combination of targets in a particular subset size. "Predict partitions" produces a list which correlates the results for a specified combination of targets with the partition selected in column 4.

Under "Parameter to maximise", choose "Simpson's index of diversity".

The other 4 parameters can also be maximised in either a heuristic search or an exhaustive search. One or more targets defining partitions must be specified if a concordance measure is being maximised; this is not a requirement for Simpson's index of diversity.

When large datasets are being examined, a number of steps can speed up the analysis. By specifying a value in "Limit combinations per subset size to:" the heuristic will randomly select that number of combinations with the maximal parameter value for analysis, rather than all possible combinations. "Stop when parameter reaches:" can be used to abort the analysis once a certain value is reached. If it is left blank, the analysis will continue until a maximum parameter value of 1 is reached. "Show progress bar" can also be unselected which can speed up the analysis.

Click "Done".

Step 4: Heuristic analysis

AuSeTTS has identified that binary targets 5 and 9 are non-contributary to the analysis and so are excluded. Click OK:

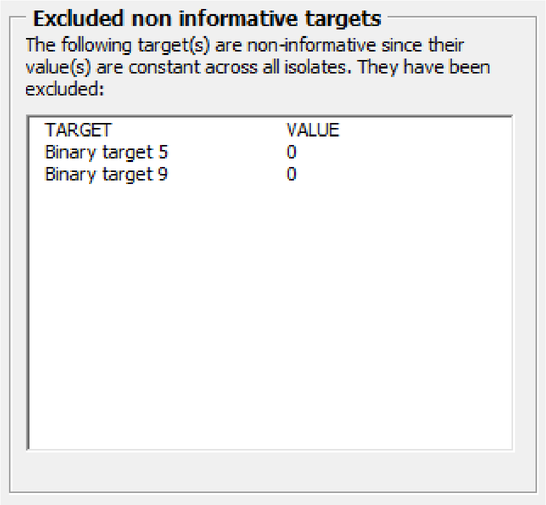

AuSeTTS has identified that Binary target 13 and Binary target 17 are fully concordant, so only one needs to be included in the analysis. Select Binary target 17 to exclude and click OK:.

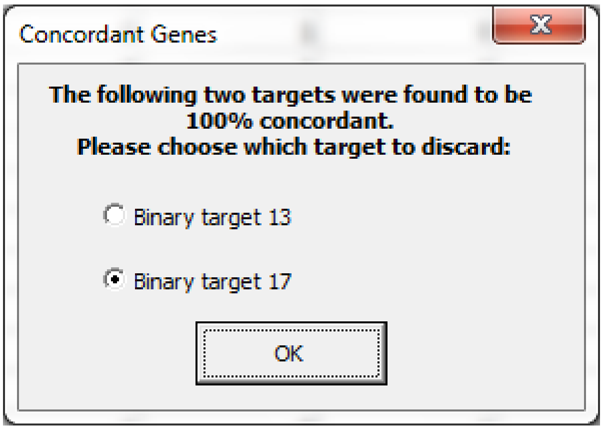

The analysis is then run, with a progress bar displaying the progress of the analysis. A warning is shown when the analysis is complete regarding the limitations of confidence intervals for the adjusted Wallace coefficient. Click OK:

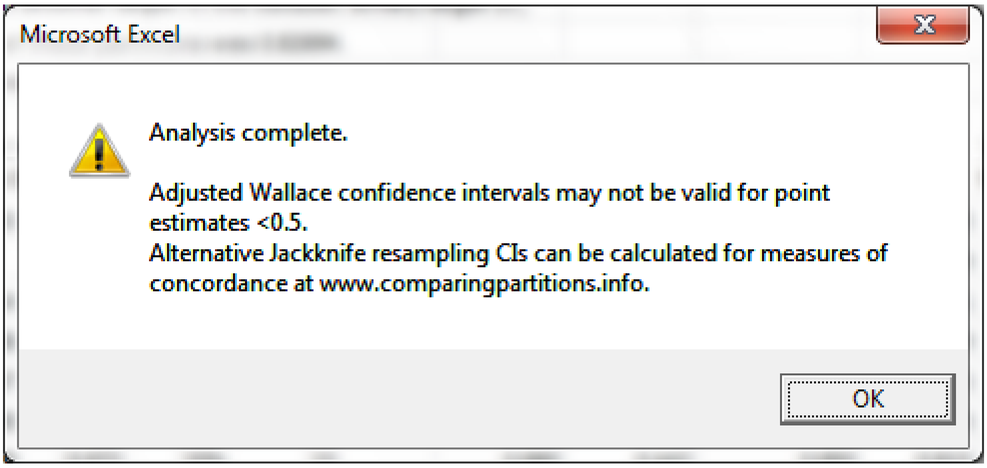

Step 5: Heuristic search results.

The results are shown on the "Results" worksheet of the AuSeTTS workbook:

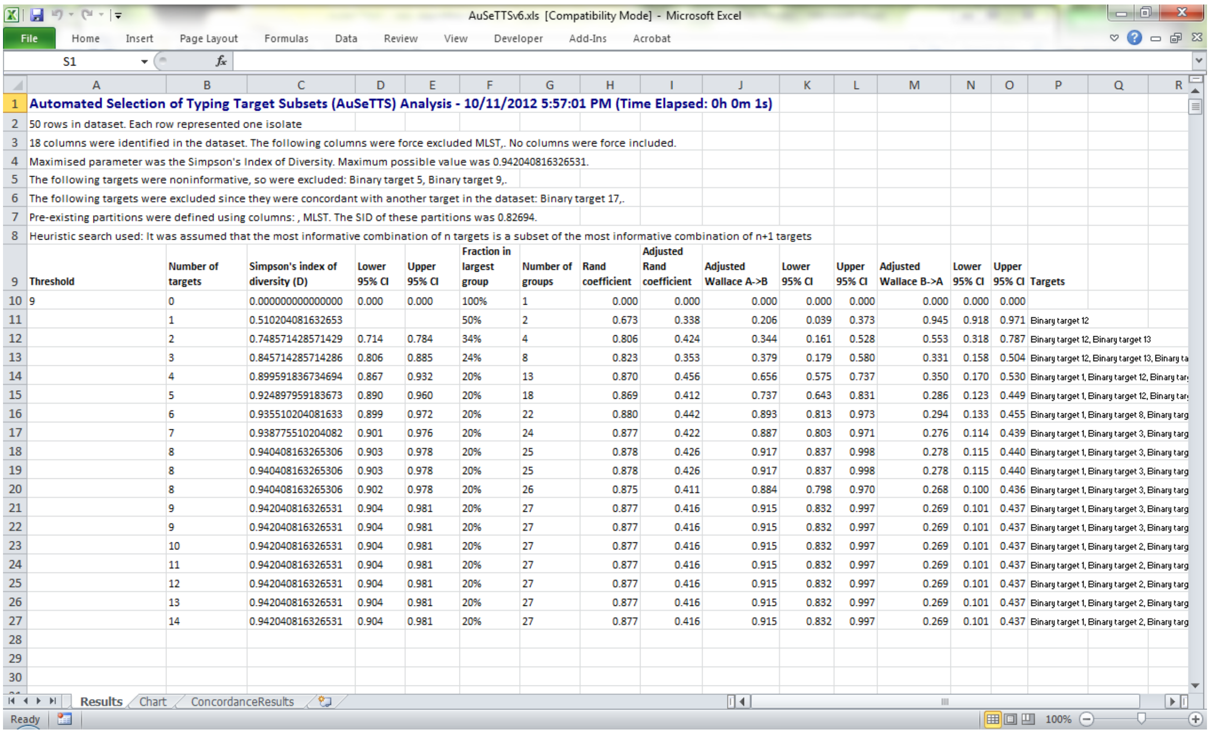

Automated Selection of Typing Target Subsets (AuSeTTS) Analysis - 10/11/2012 5:57:01 PM (Time Elapsed: 0h 0m 1s)

50 rows in dataset. Each row represented one isolate

18 columns were identified in the dataset. The following columns were force excluded MLST,. No columns were force included.

Maximised parameter was the Simpson's Index of Diversity. Maximum possible value was 0.942040816326531.

The following targets were noninformative, so were excluded: Binary target 5, Binary target 9,.

The following targets were excluded since they were concordant with another target in the dataset: Binary target 17,.

Pre-existing partitions were defined using columns: , MLST. The SID of these partitions was 0.82694.

Heuristic search used: It was assumed that the most informative combination of n targets is a subset of the most informative combination of n+1 targets

Threshold	Number of targets	Simpson's index of diversity (D)	Lower 95% CI	Upper 95% CI	Fraction in largest group	Number of groups	Rand coefficient	Adjusted Rand coefficient	Adjusted Wallace A->B	Lower 95% CI	Upper 95% CI	Adjusted Wallace B->A	Lower 95% CI	Upper 95% CI	Targets
9	0	0.000000000000000	0.000	0.000	100%	1	0.000	0.000	0.000	0.000	0.000	0.000	0.000	0.000	
	1	0.510204081632653			50%	2	0.673	0.338	0.206	0.039	0.373	0.945	0.918	0.971	Binary target 12
	2	0.748571428571429	0.714	0.784	34%	4	0.806	0.424	0.344	0.161	0.528	0.553	0.318	0.787	Binary target 12, Binary target 13
	3	0.845714285714286	0.806	0.885	24%	8	0.823	0.353	0.379	0.179	0.580	0.331	0.158	0.504	Binary target 12, Binary target 13, Binary ta
	4	0.899591836734694	0.867	0.932	20%	13	0.870	0.456	0.656	0.575	0.737	0.350	0.170	0.530	Binary target 1, Binary target 12, Binary tar
	5	0.924897959183673	0.890	0.960	20%	18	0.869	0.412	0.737	0.643	0.831	0.286	0.123	0.449	Binary target 1, Binary target 12, Binary tar
	6	0.935510204081633	0.899	0.972	20%	22	0.880	0.442	0.893	0.813	0.973	0.294	0.133	0.455	Binary target 1, Binary target 8, Binary targ
	7	0.938775510204082	0.901	0.976	20%	24	0.877	0.422	0.887	0.803	0.971	0.276	0.114	0.439	Binary target 1, Binary target 3, Binary targ
	8	0.940408163265306	0.903	0.978	20%	25	0.878	0.426	0.917	0.837	0.998	0.278	0.115	0.440	Binary target 1, Binary target 3, Binary targ
	8	0.940408163265306	0.903	0.978	20%	25	0.878	0.426	0.917	0.837	0.998	0.278	0.115	0.440	Binary target 1, Binary target 3, Binary targ
	8	0.940408163265306	0.902	0.978	20%	26	0.875	0.411	0.884	0.798	0.970	0.268	0.100	0.436	Binary target 1, Binary target 3, Binary targ
	9	0.942040816326531	0.904	0.981	20%	27	0.877	0.416	0.915	0.832	0.997	0.269	0.101	0.437	Binary target 1, Binary target 3, Binary targ
	9	0.942040816326531	0.904	0.981	20%	27	0.877	0.416	0.915	0.832	0.997	0.269	0.101	0.437	Binary target 1, Binary target 3, Binary targ
	10	0.942040816326531	0.904	0.981	20%	27	0.877	0.416	0.915	0.832	0.997	0.269	0.101	0.437	Binary target 1, Binary target 2, Binary targ
	11	0.942040816326531	0.904	0.981	20%	27	0.877	0.416	0.915	0.832	0.997	0.269	0.101	0.437	Binary target 1, Binary target 2, Binary targ
	12	0.942040816326531	0.904	0.981	20%	27	0.877	0.416	0.915	0.832	0.997	0.269	0.101	0.437	Binary target 1, Binary target 2, Binary targ
	13	0.942040816326531	0.904	0.981	20%	27	0.877	0.416	0.915	0.832	0.997	0.269	0.101	0.437	Binary target 1, Binary target 2, Binary targ
	14	0.942040816326531	0.904	0.981	20%	27	0.877	0.416	0.915	0.832	0.997	0.269	0.101	0.437	Binary target 1, Binary target 2, Binary targ

These results show that the maximum Simpson's index of diversity is 0.942040816326531 and this could be achieved with a combination of 9 binary targets (the threshold, cell A10). There were two combinations of 9 targets that could produce this value, and they are listed in cells P21 and P22:

Binary target 1, Binary target 3, Binary target 6, Binary target 8, Binary target 12, Binary target 13, Binary target 14, Binary target 15, Binary target 16

Binary target 1, Binary target 3, Binary target 8, Binary target 11, Binary target 12, Binary target 13, Binary target 14, Binary target 15, Binary target 16

Confidence intervals for Simpson's index of diversity are also shown, as well as other measures of diversity – the fraction in the largest group and the number of groups. Since a reference partition was also specified (MLST), measures of concordance are also displayed for each combination of targets listed. These include the Rand, adjusted Rand and adjusted Wallace coefficients. Adjusted Wallace A>B indicates the ability for the given combination of binary targets to predict the MLST type; Adjusted Wallace B>A indicates the ability for the MLST type to predict the indicated combination of binary targets.

The results are also shown graphically on the “Chart” worksheet:

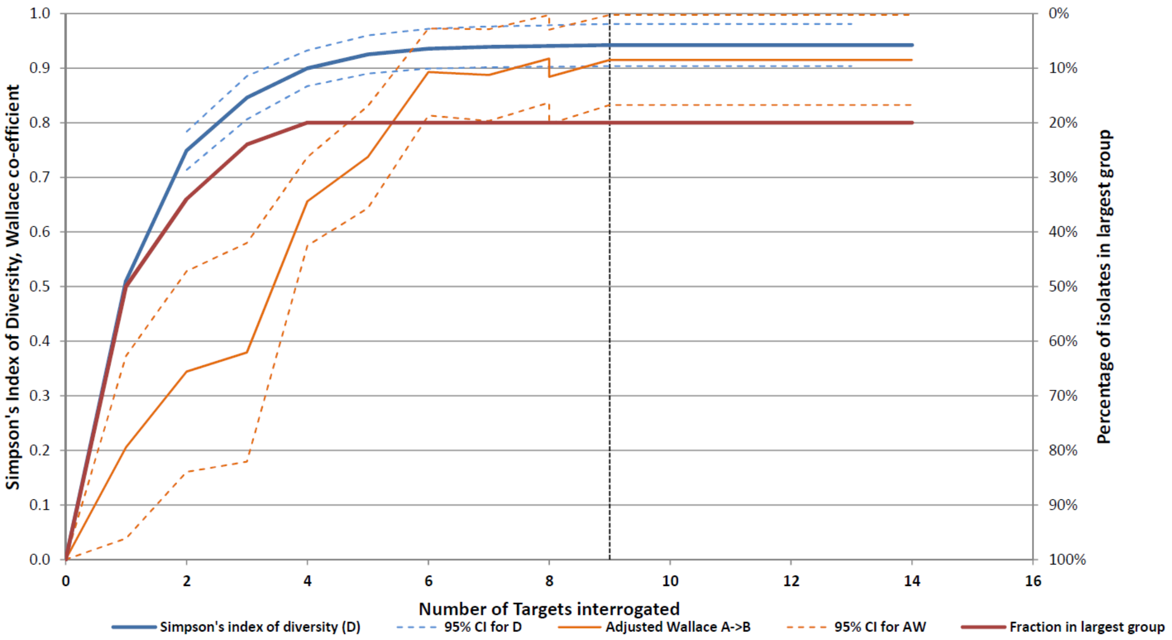

To save the results of the analyses, save the worksheet with a different name, using the “Save As…” in the Excel File menu.

Step 6: Exhaustive search

The heuristic search indicates that a combination of 9 targets is the smallest subset that will maximise the Simpson’s index of diversity. This can now be verified or refuted using an exhaustive search for a combination of 8 targets to see if subset size can provide the maximum Simpson’s index of diversity.

Press Ctrl-M to launch the AuSeTTS program again.

This time choose “Use previously loaded data” on the first popup screen.

On the “Define Fields screen”, choose “Exhaustive search” as the “Mode” and “Simpson’s index of diversity” as the parameter to maximise. Highlight “MLST” in the list of “Target(s) to Force Exclude”:

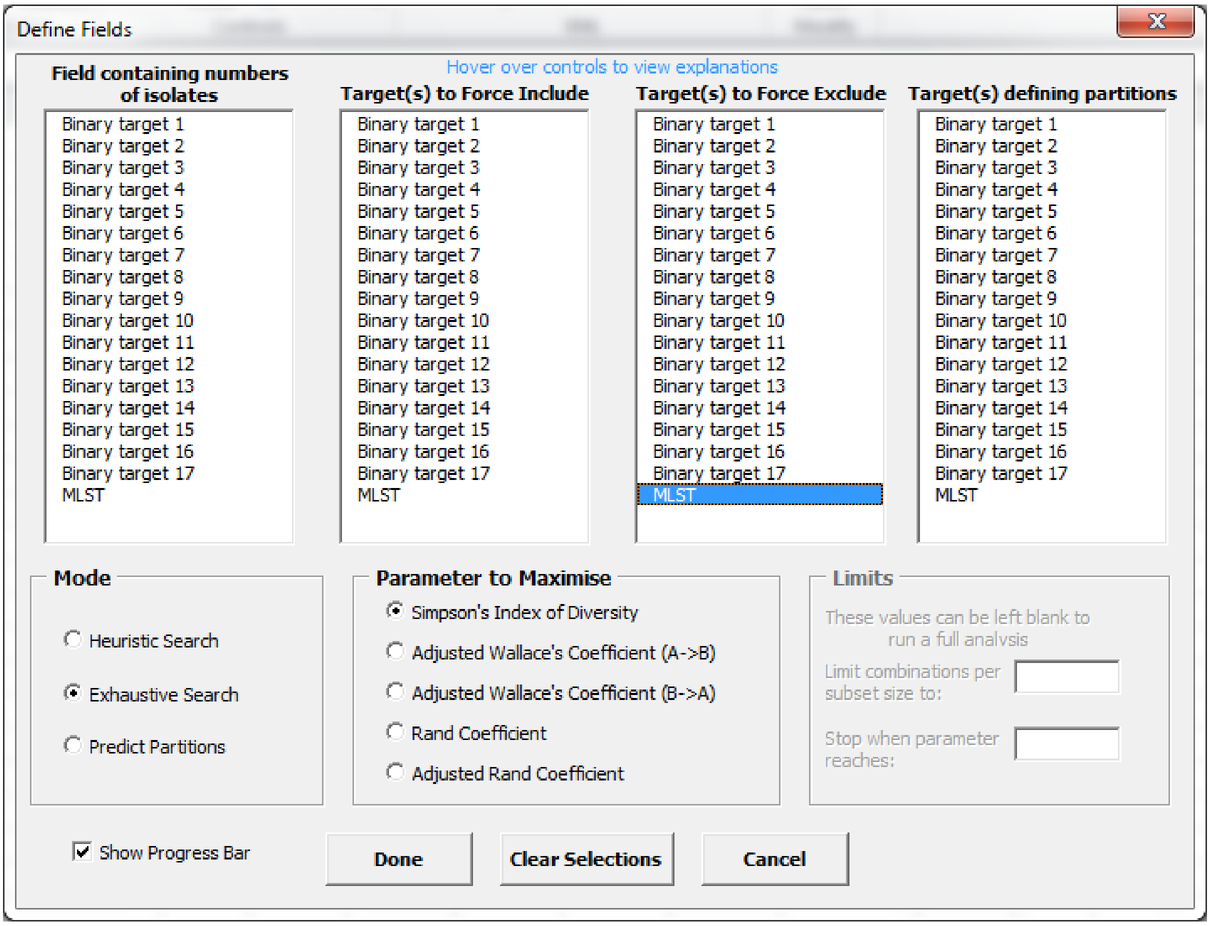

If measuring concordance with a reference partition is not required, then force exclude the targets which represent the reference partition, rather than highlighting them in the fourth window. This will speed up the analysis.

Click "Done". Again, the non informative targets are listed. Click "OK":

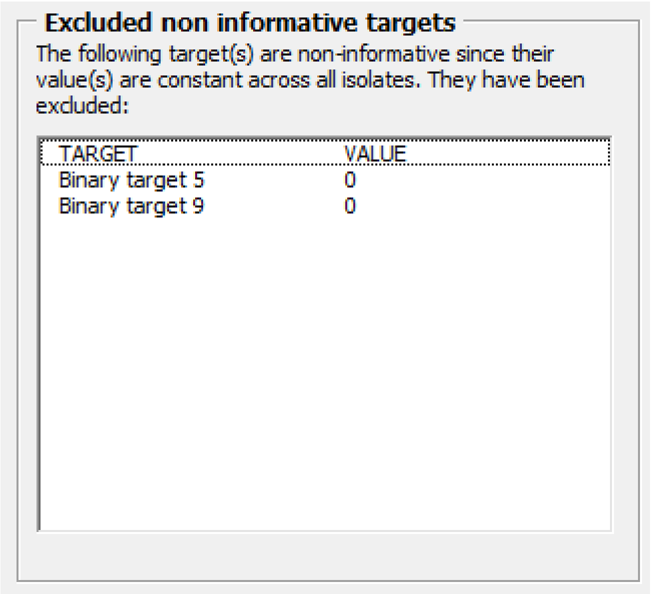

Again, the user is prompted to discard one of the concordant targets. Choose “Binary target 17” and click “OK”:

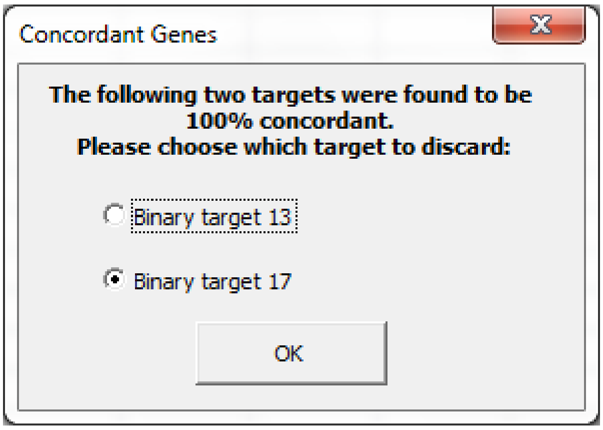

You are then prompted to choose the combination size to be examined. Since the heuristic search showed a threshold value of 9, choose one size less (ie: 8) to be examined in the exhaustive search:

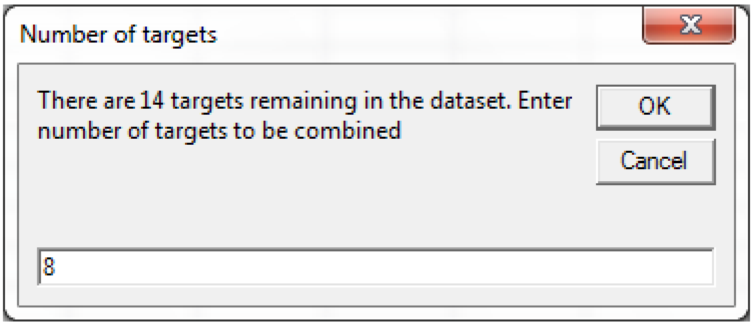

You are warned about the analysis time. Click “OK”

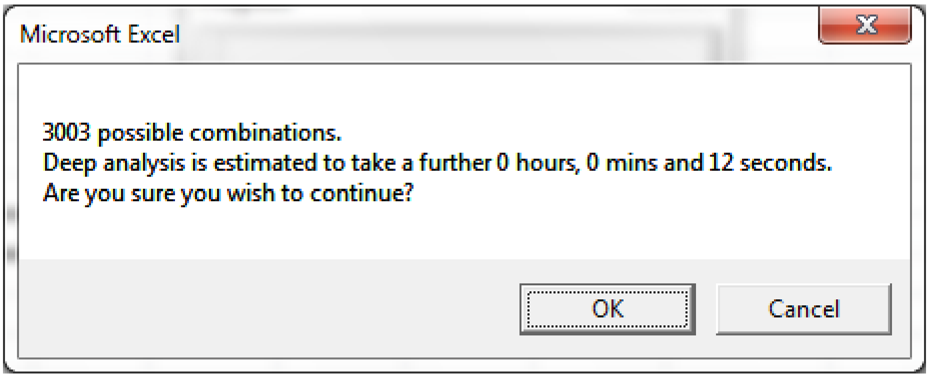

The analysis is then undertaken, and once complete, a warning about the confidence intervals for the *AW* coefficient is displayed. Click OK.

Step 7: Results of exhaustive search

The exhaustive search indicates that the maximum *D* value achievable with a combination of 8 targets is 0.940408163265306 (cell C10). Four different combinations of 8 targets was able to achieve this value (cells P10-P13). This D value is less than the maximum *D* value determined by the heuristic search, so the heuristic is found to be valid:

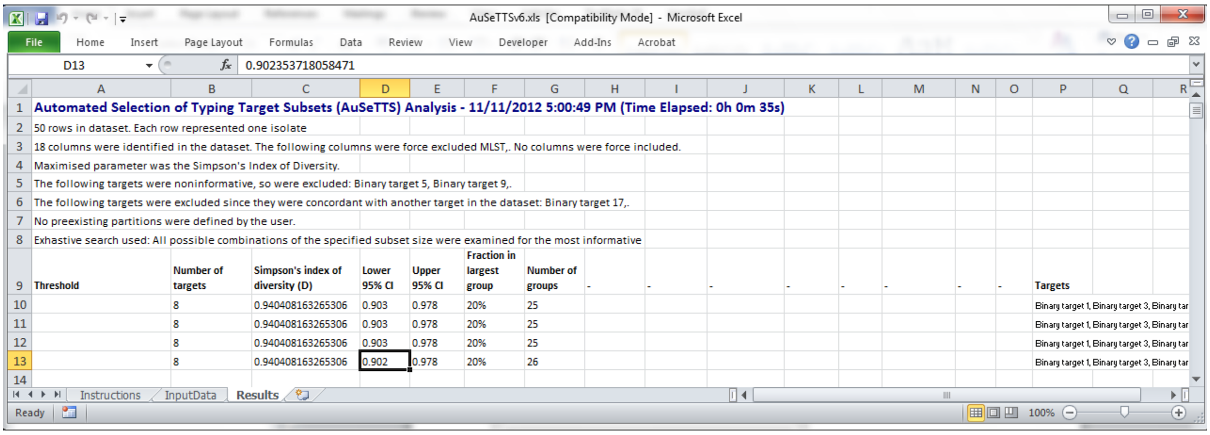

AuSeTTSv6.xls [Compatibility Mode] - Microsoft Excel

D13 | 0.902353718058471

Automated Selection of Typing Target Subsets (AuSeTTS) Analysis - 11/11/2012 5:00:49 PM (Time Elapsed: 0h 0m 35s)

50 rows in dataset. Each row represented one isolate

18 columns were identified in the dataset. The following columns were force excluded MLST,. No columns were force included.

Maximised parameter was the Simpson's Index of Diversity.

The following targets were noninformative, so were excluded: Binary target 5, Binary target 9,.

The following targets were excluded since they were concordant with another target in the dataset: Binary target 17,.

No preexisting partitions were defined by the user.

Exhastive search used: All possible combinations of the specified subset size were examined for the most informative

Threshold	Number of targets	Simpson's index of diversity (D)	Lower 95% CI	Upper 95% CI	Fraction in largest group	Number of groups	-	-	-	-	-	-	-	-	Targets
	8	0.940408163265306	0.903	0.978	20%	25									Binary target 1, Binary target 3, Binary tar
	8	0.940408163265306	0.903	0.978	20%	25									Binary target 1, Binary target 3, Binary tar
	8	0.940408163265306	0.903	0.978	20%	25									Binary target 1, Binary target 3, Binary tar
	8	0.940408163265306	0.902	0.978	20%	26									Binary target 1, Binary target 3, Binary tar

Instructions | InputData | Results

Columns H through O are blank since concordance measures were not assessed in this analysis.

Choose "Save As…" from the file menu to save the results of this analysis if required.
